# Regulation and Possible Functions of Kisspeptin in the Medial Amygdala

**DOI:** 10.3389/fendo.2017.00191

**Published:** 2017-08-07

**Authors:** Shannon B. Z. Stephens, Alexander S. Kauffman

**Affiliations:** ^1^Department of Reproductive Medicine, University of California, San Diego, La Jolla, CA, United States

**Keywords:** kisspeptin, *Kiss1*, Kiss1r, amygdala, GnRH, LH, reproduction, puberty

## Abstract

Kisspeptin, encoded by the *Kiss1* gene, is required for reproduction. Humans and mice lacking kisspeptin or its receptor, Kiss1r, have impairments in reproductive physiology and fertility. In addition to being located in the hypothalamus in the anteroventral periventricular and arcuate nuclei, kisspeptin neurons are also present in several extra-hypothalamic regions, such as the medial amygdala (MeA). However, while there has been a significant focus on the reproductive roles of hypothalamic kisspeptin neurons, the regulation and function(s) of MeA and other extra-hypothalamic kisspeptin neurons have received far less attention. This review summarizes what is currently known about the regulation, development, neural projections, and potential functions of MeA kisspeptin neurons, as well as kisspeptin signaling directly within the MeA, with emphasis on data gathered from rodent models. Recent data are summarized and compared between rodent species and also between males and females. In addition, critical gaps in knowledge and important future directions are discussed.

## Introduction

Kisspeptin, encoded by the *Kiss1* gene, is essential for reproduction. Humans and mice lacking *Kiss1* or its receptor, *Kiss1r*, have deficits in puberty onset, reproductive hormone release, and fertility (1–4). In humans and rodents, kisspeptin treatment directly stimulates GnRH neurons to increase downstream LH and FSH secretion (5–12). Kisspeptin-synthesizing neurons are primarily located in two regions of the hypothalamus, the anteroventral periventricular (AVPV)/rostral periventricular (PeN) continuum and the arcuate nucleus (ARC) (9, 13–17). The AVPV/PeN and ARC *Kiss1* populations are differentially regulated by testosterone (T) and estradiol (E_2_). In the AVPV/PeN, E_2_ increases *Kiss1* levels and gonadectomy (GDX) decreases *Kiss1* levels, supporting the proposed role of these neurons in mediating E_2_-positive feedback induction of the pre-ovulatory LH surge in females (13–15). Supporting this, *Kiss1* neurons are sexually differentiated, being more numerous, and expressing higher *Kiss1* mRNA levels in females (15, 18). By contrast, *Kiss1* expression in the ARC increases following GDX and decreases with T or E_2_ treatment. Thus, ARC *Kiss1* neurons are thought to participate in gonadal steroid negative feedback and the pulsatile release of GnRH secretion (13–15).

Smaller populations of *Kiss1* neurons have also recently been identified in several extra-hypothalamic areas, including the medial amygdala (MeA), bed nucleus of the stria terminalis (BnST), and lateral septum (9, 19–23). However, these extra-hypothalamic neurons have not been studied extensively, and their regulation and functions are only now beginning to be determined. In particular, the MeA region, part of the limbic system, is known to have numerous behavioral and physiological functions, including (but not limited to) roles in sexual behavior and reproductive physiology (24–31). This review summarizes what is currently known about the regulation, development, and function(s) of MeA kisspeptin neurons as well as kisspeptin signaling directly within the MeA.

## Identification and Regulation of Kisspeptin Neurons in the MeA

**Figure 1:**
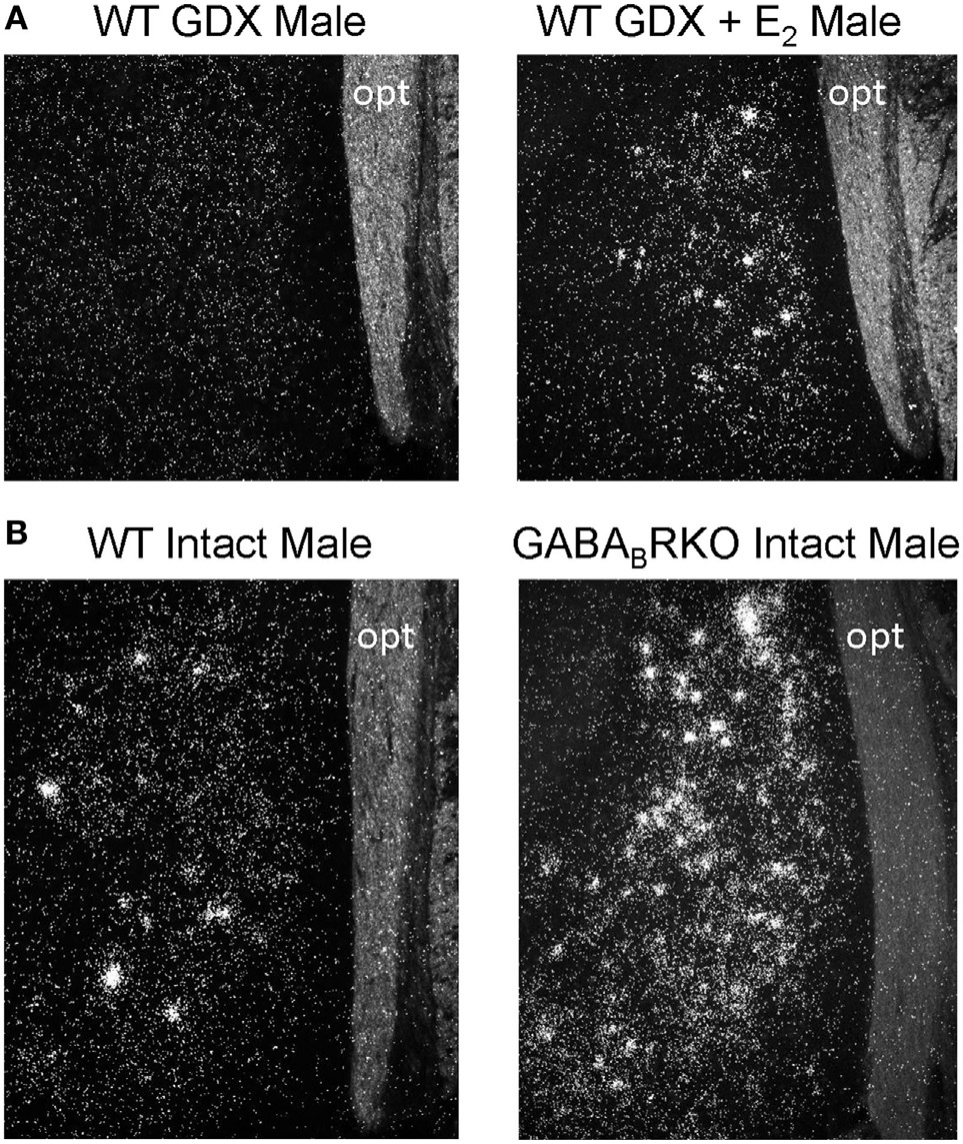
Medial amygdala (MeA) *Kiss1* expression (silver grains in *in situ* hybridization photomicrographs) is regulated by E_2_ and GABA signaling. **(A)** Gonadectomized (GDX) mice have few, if any, *Kiss1* cells in the MeA, whereas E_2_ treatment significantly increases MeA *Kiss1* expression. **(B)** Gonad-intact GABA_B_R KO mice have substantially more MeA *Kiss1* than gonad-intact wild-type (WT) males, despite comparable circulating sex steroid levels. opt, optic tract.

*Kiss1* neurons in the MeA were first observed in male mice in 2004 (9) but were not directly studied until 2011 when Kim et al. first tested whether rodent MeA *Kiss1* neurons are regulated by sex steroids (19), as occurs for hypothalamic kisspeptin neurons. The authors found that, as in the AVPV/PeN, *Kiss1* levels in the MeA are strongly upregulated by sex steroids in both mice and rats. Specifically, in adult, gonadectomized (GDX) mice and rats, there are few, if any, detectable MeA *Kiss1* cells, whereas exogenous treatment with T or E_2_ significantly increases MeA *Kiss1* cell number (19) (Figure 1A). Unlike E_2_ treatment, DHT treatment did not increase MeA *Kiss1* expression, indicating the sex steroid upregulation of MeA *Kiss1* occurs through estrogen receptors (ERs) rather than androgen receptors (19). Xu and colleagues (21) similarly determined that exogenous E_2_ treatment in male and female rats upregulates both MeA *Kiss1* mRNA and MeA kisspeptin protein expression (21).

The MeA region is known to express high levels of sex steroid receptors, including both ERα and ERβ (the latter is not highly expressed in the ARC or AVPV/PeN), but it was not initially known which ER mediates E_2_ stimulation of MeA *Kiss1* neurons. Recent data from ERαKO mice indicate that both the hypothalamic (13, 14, 32, 33) and MeA (33) *Kiss1* cells are primarily regulated *via* ERα. In the ARC and AVPV/PeN, E_2_ treatment alters *Kiss1* expression in wild-type (WT) mice but not in ERαKO mice (13, 14, 32, 33). Similarly, in the MeA, E_2_ robustly increases *Kiss1* expression in WT mice, whereas ERαKO mice given E_2_ failed to show comparable large increases in MeA *Kiss1* levels (33). Thus, substantial E_2_ stimulation of MeA *Kiss1* requires ERα signaling. However, E_2_-treated ERαKO mice did show a minor increase in MeA *Kiss1* levels compared with non-E_2_-treated ERαKOs, indicating that another ER may partially compensate for the loss of ERα. This partial increase in *Kiss1* expression in E_2_-treated ERαKO mice is unique to the MeA, as hypothalamic *Kiss1* levels in E_2_-treated ERαKOs were comparable to that of non-E_2_-treated ERαKOs (33). By contrast, *Kiss1* expression in the MeA (and hypothalamus) of ERβKO mice of both sexes mirrored that of WT mice under all hormonal conditions (13, 33). Thus, unlike ERα, ERβ is not required for E_2_’s regulation of either hypothalamic or MeA *Kiss1*.

In the MeA of gonad-intact rats and mice, *Kiss1* expression is higher in males than in diestrus females (19). However, gonad-intact female rats have increased MeA *Kiss1* expression during proestrus (when circulating E_2_ levels are highest), and relatively low levels during estrus and diestrus (19). Thus, the sex difference in MeA *Kiss1* expression between gonad-intact males and females is likely due to differences in circulating sex steroid levels. Indeed, when E_2_ levels are equalized between males and females, the previously observed sex difference in MeA *Kiss1* levels disappears, with males and females now showing comparable elevated MeA *Kiss1* (19, 21) and kisspeptin (21) expression.

In addition to E_2_, GABA signaling *via* GABA_B_R also strongly regulates MeA *Kiss1* expression. In gonad-intact GABA_B_R KO mice, MeA *Kiss1* levels are drastically elevated in comparison to WT mice (22) (Figure 1B). This large increase in MeA *Kiss1* expression is not due to altered sex steroid levels, as circulating T (males) or E_2_ (females) were similar between WT and GABA_B_R KOs (22). Interestingly, the elevated *Kiss1* levels in the MeA of GABA_B_R KOs are typically much greater than observed for E_2_ stimulation of *Kiss1* in this region in WT mice. This suggests that endogenous GABA_B_R signaling is a very potent regulator of MeA *Kiss1*. Interestingly, this large upregulation of *Kiss1* levels in GABA_B_R KO mice was exclusive to extra-hypothalamic *Kiss1* expression, as AVPV and ARC *Kiss1* expression were normal in GABA_B_R KO mice (22). Thus, GABA signaling *via* GABA_B_R may normally serve to inhibit MeA *Kiss1* expression, whereas GABA_B_R signaling seems to have no major effect on hypothalamic *Kiss1* levels. Whether this observed effect is due to GABA_B_R signaling directly in MeA *Kiss1* neurons remains to be determined, though MeA *Kiss1* neurons do express GABA_B_R (22).

## Developmental Expression of Kisspeptin Neurons in the MeA

Although MeA *Kiss1* expression is detectable in adult rodents, especially when sex steroids are elevated, MeA *Kiss1* expression is not detected in prepubertal rodents, at postnatal day (PND) 14 in mice (22) or PND 19 and earlier in rats (34). Similarly, MeA *Kiss1* expression was absent at PND 14 in GABA_B_R KO mice despite being dramatically elevated in these mice in adulthood (22). These findings indicate that in juvenile and prepubertal rodents either (1) MeA *Kiss1* neurons are not yet present or (2) MeA *Kiss1* neurons are present but not able to express *Kiss1*. The latter possibility might reflect the absence of elevated circulating sex steroids before puberty, therefore precluding notable MeA *Kiss1* expression at young ages.

The developmental pattern of MeA *Kiss1* expression was recently examined every 5 days, from PND 15 until PND 40, in gonad-intact C57BL6 male mice, with puberty occurring around PND 35 (33). MeA *Kiss1* was first detected at very low levels around PND 20–25, but did not significantly increase until PND 35, with highest expression at PND 40 (the oldest age examined). Circulating T levels in the same mice mirrored the developmental pattern of MeA *Kiss1* expression, similarly increasing at PND 35 (as expected with puberty) (33). However, whether the pubertal increase in T caused or resulted from the increase in MeA *Kiss1* expression is unclear. Therefore, the second study treated juvenile (PND 14) male mice with high-dose E_2_ for 4 days to determine if elevated E_2_ exposure at this prepubertal age could prematurely increase MeA *Kiss1* expression (33). Indeed, juvenile E_2_ treatment increased MeA *Kiss1* at PND 18 versus untreated controls, demonstrating that by PND 18, MeA *Kiss1* neurons are present and capable of expressing notable *Kiss1 if* sex steroids are sufficiently elevated (33). Thus, increases in MeA *Kiss1* expression around puberty are likely a response to rising circulating sex steroid levels at this time. Whether this emergence of notable MeA *Kiss1* levels at puberty is functionally relevant to the pubertal process or to other physiological/behavioral processes is currently unknown.

## MeA Kisspeptin Neuron Projections and Possible Functions

Understanding where MeA kisspeptin neurons project to can inform upon their potential functions. Research examining the afferent and efferent projections of MeA kisspeptin neurons only recently began, and thus, little is currently known. Using double-label immunohistochemistry, Pineda and colleagues demonstrated in male rats that some MeA kisspeptin neurons receive neural appositions from “upstream” neurons containing tyrosine hydroxylase (TH), the rate-limiting enzyme in catecholamine synthesis, and vasopressin (35). 25 and 11% of MeA kisspeptin neurons receive inputs from TH and vasopressin neurons, respectively (35). However, it is currently unknown if MeA kisspeptin neurons express either dopamine receptors or vasopressin receptors or from where in the brain the dopamine or vasopressin signaling originates. It is also unknown whether dopamine or vasopressin alters MeA *Kiss1* or kisspeptin expression or neuronal activity, and if so, whether such regulation is positive or negative. Regardless, these data suggest that MeA kisspeptin neurons may have a role in participating in dopamine- or vasopressin-dependent physiology/behaviors, such as motivation and reward-seeking behaviors or social behaviors.

In rodents, the MeA has been implicated in regulating reproductive physiology because MeA lesions in rats disrupt ovarian cycles and alter pubertal timing (24–26, 31, 36–38). However, the specific cell types in the MeA that influence the reproductive axis are unknown. Given kisspeptin’s potent actions on GnRH neurons, *Kiss1* neurons in the MeA are good candidates to serve as reproductive signalers from this brain region. Supporting this possibility, in female mice, MeA kisspeptin neurons send axonal projections to the preoptic area (POA), where many GnRH neurons reside (23). Moreover, injections of an AAV-DIO-YFP virus into the MeA of male KissCre-GFP mice revealed that ~15% of GnRH neurons in the POA receive close fiber appositions from MeA kisspeptin neurons (35). These anatomical data suggest that MeA kisspeptin neurons may have the potential to modulate a subset of GnRH neurons, though it should be noted that *most* GnRH neurons did not receive MeA kisspeptin contacts (which may be a result of technical/methodological limitations). Additional studies are therefore needed to determine if—and to what degree—MeA kisspeptin cells project to GnRH neurons.

Anterograde tracing studies in male rats recently demonstrated that the accessory olfactory bulb (AOB), but not the main olfactory bulb, projects to MeA kisspeptin neurons (35). This suggests that MeA kisspeptin neurons may also have a role in processing or responding to olfactory/pheromone cues, potentially social signals. Supporting this, selective chemogenetic activation of MeA kisspeptin neurons in male mice increased the amount of time males spent investigating estrous females (39). In that study, male KissCre-GFP mice received bilateral injections of a stimulatory viral DREADD receptor construct into the posterodorsal MeA, followed 4 weeks later with peripheral injection of clozapine-N-oxide (CNO) to selectively activate MeA kisspeptin neurons (39). Although selective DREADD activation of MeA kisspeptin neurons increased males’ investigation of females, it also increased the amount of time males spent with juvenile conspecifics (39), indicating that MeA kisspeptin neurons may modulate behavioral responses to any social odors, not just opposite-sex odors. Importantly, it remains unknown whether these induced behavioral changes are due to kisspeptin or another neuropeptide/neurotransmitter co-released from MeA kisspeptin neurons when activated. A prior study in *Kiss1r* KO mice showed that kisspeptin signaling is required for proper opposite-sex odor preference (40). Thus, it may be specifically kisspeptin (rather than another signaling factor) from these MeA neurons that are modulating opposite-sex and juvenile odor preference, but this still needs to be determined. Interestingly, in addition to receiving afferent projections from the AOB, MeA kisspeptin neurons also send reciprocal projections back to the AOB region, specifically the mitral and granule layers in mice and the granule layer in rats (23, 35). Mitral and granule cells are part of a reciprocal feedback circuit, with mitral cells exciting granule cells *via* glutamate and granule cells inhibiting mitral cells *via* GABA signaling (41–46). The functional relevance of such MeA kisspeptin signaling to the AOB cells remains to be determined.

Finally, Adekunbi and colleagues recently found that selective DREADD activation of MeA kisspeptin neurons reduces anxiety, with CNO-treated mice spending more time exploring the open arms in an elevated-plus maze than control mice. This suggests that MeA kisspeptin neurons may lower anxiety-related behaviors. However, these results differ from previous data showing intracerebroventricular (icv) injection of kisspeptin-13 in male rats decreased time spent in the open arms of the elevated-plus maze, suggesting that kisspeptin increased anxiety (47). These conflicting findings may reflect species differences or different methodologies (i.e., selective DREADD activation of just MeA kisspeptin neurons versus increased kisspeptin signaling throughout the brain). It is also possible that another neuropeptide/neurotransmitter released from MeA kisspeptin neurons caused the anxiolytic behavior in the DREADD study. Additional research is needed to clarify the role of MeA kisspeptin neurons in modulating anxiety.

## Kisspeptin Signaling within the MeA

Thus far, this review has focused on the regulation, projections, and functions of kisspeptin neurons residing in the MeA, but several studies have also examined the role of kisspeptin signaling within the MeA. Intra-MeA injections of kisspeptin-10 in GDX + E_2_ (diestrus E_2_ levels) female rats dose dependently increased LH within an hour (48). Thus, kisspeptin acting directly within the MeA may also modulate GnRH release. Supporting this hypothesis, intra-MeA injection of a kisspeptin antagonist, peptide-234, in GDX + E_2_-treated females decreased LH secretion 2–4 h later (48). This suggests that *endogenous* kisspeptin signaling acting within the MeA is important for normal GnRH/LH secretion. Although it is possible that the kisspeptin or antagonist treatment spread outside of the MeA to other brain areas, other studies showed that icv injections of this same dose, 100 pmol, had no effect on LH levels (49, 50). Therefore, in female rats, kisspeptin may act directly within the MeA to modulate GnRH/LH release. In male rats, a similar dose of 100 pmol of kisspeptin-10 injected directly into the MeA also increased LH levels (51), with a greater LH increase after a higher dose, 1 nmol (51). Peptide-234 injected directly into the MeA of male rats did not alter LH (51), unlike in females, which may reflect a sex difference in endogenous MeA kisspeptin signaling targeting the MeA. In addition to increasing LH, intra-MeA injections of kisspeptin-10 also increased ex-copula erections in rats, which was prevented by concurrent kisspeptin antagonist treatment (51). Thus, kisspeptin signaling in the MeA of male rodents may regulate reproductive physiology and behavior.

The functional consequences of kisspeptin signaling directly within the MeA discussed above have only been examined in rats, for which there is some evidence of kisspeptin receptor, *Kiss1r*, in the MeA. Radiolabeled *in situ* hybridization found abundant expression of *Kiss1r* in the rat amygdala (52). However, non-radioactive ISH found no *Kiss1r* expression in the MeA (53), perhaps because non-radioactive ISH is less sensitive and, therefore, unable to detect low *Kiss1r* expression (53). *Kiss1r* expression has not been detected in the MeA of mice (54), indicating the functions of kisspeptin signaling acting in the MeA may be species dependent, though further studies on this issue are needed.

If kisspeptin can in fact act directly in the MeA, then where is such kisspeptin signaling coming from? It is currently unknown if any kisspeptin neuronal population, hypothalamic or extra-hypothalamic, projects to the MeA to be the potential source of kisspeptin acting in this area. One possibility could be that MeA kisspeptin neurons project locally, within the MeA, to regulate other neurons in this region, but this has not been studied. Additional research is therefore needed to determine if MeA-derived kisspeptin acts locally within the MeA or if kisspeptin action within the MeA is due to kisspeptin release from other areas.

## Gaps in Knowledge and Future Directions

**Figure 2:**
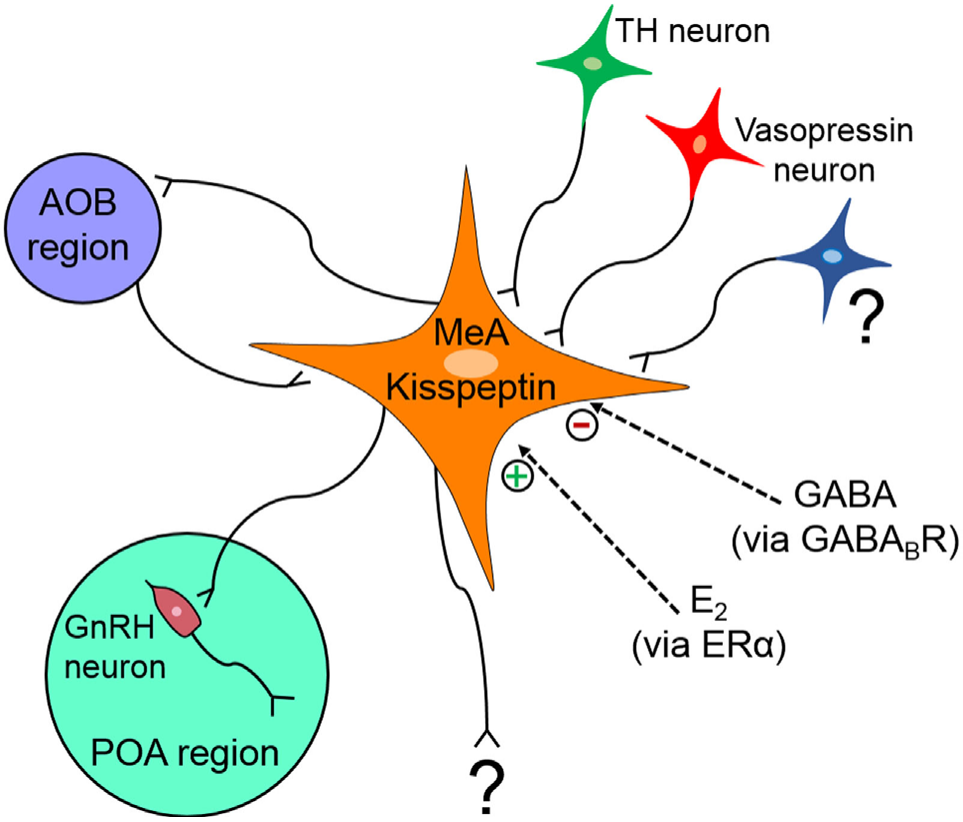
Schematic diagram summarizing what is known about the regulation and projections of medial amygdala (MeA) kisspeptin neurons. MeA kisspeptin neurons receive projections from tyrosine hydroxylase (TH) and vasopressin neurons, as well as from neurons originating in the accessory olfactory bulb (AOB). MeA kisspeptin neurons have efferent projections back to the AOB and also to some GnRH neurons in the preoptic area (POA). MeA *Kiss1* neurons are upregulated by E_2_ *via* ERα and downregulated by GABA signaling *via* GABA_B_R, but it is currently unknown if this E_2_ and GABA regulation occurs directly on MeA *Kiss1* cells or indirectly via intermediary neurons. ? = unknown afferent or efferent projections of MeA kisspeptin neurons.

The MeA region is implicated in many diverse functions and behavioral processes, and deciphering the specific function(s) of kisspeptin neurons in this region is therefore not simple. Figure 2 summarizes our current understanding of the regulation and function(s) of MeA kisspeptin neurons. Although MeA kisspeptin neurons are clearly regulated by E_2_ and GABA signaling, *via* ERα and GABA_B_R, respectively, whether this regulation occurs directly or indirectly on MeA kisspeptin neurons is unknown. Supporting a possible direct regulation, MeA kisspeptin neurons express GABA_B_R, and ERα is heavily expressed in the MeA region (though ERα specifically in MeA kisspeptin neurons has not been determined). TH and vasopressin neurons project to MeA kisspeptin neurons; however, whether MeA kisspeptin neurons express dopamine or vasopressin receptors is unknown, as is any effect of dopamine or vasopressin on MeA kisspeptin neurons. Additional research is also needed to determine what other factors may regulate MeA kisspeptin neurons. Other than kisspeptin, it is also currently unknown what other signaling factors are produced by MeA *Kiss1* neurons. This is important for knowing whether the LH or behavioral responses following DREADD activation of these neurons are due to kisspeptin or some other co-released neuropeptide/neurotransmitter.

MeA *Kiss1* expression is first detected around puberty, when gonadal steroids are also rising. Data suggest that the increase in MeA *Kiss1* at this time is likely caused by the pubertal increases in gonadal sex steroids, but this requires further examination. Regardless, the presence of notable *Kiss1* in the MeA only at puberty and beyond suggests that the functional relevance of kisspeptin released from the MeA is restricted to processes during sexual maturation and/or adulthood.

MeA kisspeptin neurons send axon projections to some GnRH neurons, which may indicate MeA kisspeptin neurons can modulate the reproductive axis. This is supported by LH increases after MeA *Kiss1* neuron DREADD activation. Lesions of the entire MeA disrupt ovarian cycles and alter puberty, perhaps because of ablated MeA kisspeptin neurons, but this has not been studied. MeA kisspeptin neurons also form a reciprocal circuit with the AOB, and activation of MeA kisspeptin neurons increases social interactions in mice, indicating MeA kisspeptin neurons may influence social and/or sexual olfactory processing. Activation of MeA kisspeptin neurons also decreases anxiety behavior, suggesting kisspeptin or another neuropeptide/neurotransmitter released from these neurons influences anxiety. Other possible neural targets of MeA kisspeptin neurons remain to be determined and are needed to understand the functions, reproductive or otherwise, of these particular MeA neurons.

## Author Contributions

Both authors researched and wrote/edited the article and designed the figures.

## Conflict of Interest Statement

The authors declare that the research was conducted in the absence of any commercial or financial relationships that could be construed as a potential conflict of interest.
